# Contextual and psychosocial influences on antiretroviral therapy adherence in rural Zimbabwe: towards a systematic framework for programme planners

**DOI:** 10.1002/hpm.1082

**Published:** 2011-07-10

**Authors:** Morten Skovdal, Catherine Campbell, Kundai Nhongo, Constance Nyamukapa, Simon Gregson

**Affiliations:** 1 London School of Economics and Political Science, Institute of Social PsychologyLondon, UK; 2 Biomedical Research and Training InstituteHarare, Zimbabwe; 3 Imperial College London, Department of Infectious Disease and EpidemiologyLondon, UK

**Keywords:** Antiretroviral therapy, treatment, adherence, social context, HIV and AIDS, Zimbabwe

## Abstract

Great progress has been made in achieving universal access to antiretroviral therapy (ART). However, for successful viral suppression, patients must adhere to rigid and complex treatment regimens. With three quarters of antiretroviral (ARV) users in Africa adhering successfully, African countries have achieved extraordinary levels of adherence given the levels of poverty in which many ARV users live. Nevertheless, one quarter of ARV users still struggle to adhere and run the risk of experiencing viral replication, clinical progression or even drug resistance. Much has been written about ART adherence, but little has been done to systematically categorise the spectrum of factors that influence ART. In this paper, we use a Zimbabwean case study to develop a framework for ART programme planners and implementers seeking to identify and tackle social obstacles to adherence. We draw on interviews and group discussions with 25 nurses and 53 adult ARV users, which we analysed through a three-tiered thematic approach, allowing us to categorise our findings into broader dimensions that can transcend our case study and be applied elsewhere. Our findings suggest that ART adherence is influenced by the material, symbolic, relational and institutional contexts in which ARV users live as well as the patient's motivation, participation and psychosocial responses to ART. This framework allows us to examine both the social context in which ART programmes are located and the psychosocial factors that influence patient behaviours. We offer this framework as a resource for ART programme planners and implementers seeking to improve ART compliance in resource-poor settings. Copyright © 2011 John Wiley & Sons, Ltd.

## INTRODUCTION

Thanks to the planning and extraordinary management of national health departments in sub-Saharan Africa and their partners, the last couple of years have seen rapid progress in scaling up antiretroviral therapy (ART) for people living with HIV and AIDS (PLWHA) in the region (WHO *et al.*, 2009). It is estimated that 44% of Africans with advanced HIV infection are currently receiving ART (WHO, 2010). However, ART programme planners and implementers should not measure their success through the number of people receiving ART at a given time but also take into account their patients' successful compliance to treatment. The virologic efficacy of ART, or ‘good adherence’, is better achieved if patients stick to their treatment regimen for 95% of the time (Attaran, 2007). In resource-poor settings this is not easy to achieve, and many fail to adhere in the long term (Rosen *et al.*, 2007). Although much work has been done to inform ART programme planners and implementers of particular determinants and predictors of poor ART adherence, no efforts have yet been made to develop a framework that allows them to analyse the spectrum of social and behavioural factors influencing ART adherence. In this paper, we seek to contribute to the development of such an analytical framework, believing this will help ART programme planners and implementers to pinpoint possible obstacles and facilitators to adherence. To do this, we ‘mined’ our user-service interface study for any information that threw light on factors facilitating or hindering adherence. On the basis of a ‘grounded theory’ analysis of the views of ART users and providers in rural Zimbabwe—a country where no other study on ART adherence has yet been carried out—two levels of analysis emerged, namely, the contextual and psychosocial.

The ART adherence rate in sub-Saharan Africa compares favourably with that in North America (Mills *et al.*, 2006a, 2006b). However, virologic failure still occurs in settings with otherwise high levels of reported adherence (Bisson *et al.*, 2008), raising questions about how adherence is measured and monitored (Chalker *et al.* 2008). The time frame in which adherence is measured must be considered. A review of the literature, for example, found that only 60% of ART users in sub-Saharan Africa were still enrolled onto an ART programme after 2 years (Rosen *et al.*, 2007). This, coupled with less successful programmes going unreported (Gill *et al.*, 2005) and numerous studies continuing to report poor levels of adherence (Uzochukwu *et al.*, 2009), suggests that there is no room for complacency in addressing the factors that either facilitate or undermine ART adherence.

The majority of research on ART adherence in Africa is heavily focused on barriers. The common barriers identified in both qualitative and quantitative studies include stigma, treatment-related costs, distance to and waiting times at health facilities, perceptions of side effects, lack of food and inadequate knowledge of ART (Weiser *et al.*, 2003; Mshana *et al.*, 2006; Hardon *et al.*, 2007; Dahab *et al.*, 2008; Posse *et al.*, 2008; Murray *et al.*, 2009; Roura *et al.*, 2009). However, also gender (Kempf *et al.*, 2009; Uzochukwu *et al.*, 2009), alcohol use (Nachega *et al.*, 2006; Dahab *et al.*, 2008; Fitzgerald *et al.*, 2010), depression, hopelessness, psychopathology (Murray *et al.*, 2009; Adewuya *et al.*, 2010), forgetfulness, complacency (Krebs *et al.*, 2008) and dissatisfaction with hospital facilities and staff (Mshana *et al.*, 2006; Sanjobo *et al.*, 2008; Kip *et al.*, 2009) have meant that some patients do not achieve optimal adherence. Although a few of these studies refer in passing to the potential facilitators of ART adherence, only a handful of studies have looked explicitly at the facilitators of ART adherence (cf. Ware *et al.*, 2009; Watt *et al.*, 2009b). Some of the facilitators that have been identified include encouragement from peers, perceived health benefits, hope and personal motivation (Roura *et al.*, 2009), social support, treatment partners, belief in value of treatment, living up to social responsibilities, opportunities to fit ART into a daily routine (Dahab *et al.*, 2008; Ware *et al.*, 2009), feeling better, having knowledge about ART, engagement with income-generating activities (IGAs), disclosure of HIV status and praying (Sanjobo *et al.*, 2008; Watt *et al.*, 2009b).

It is clear that much information has been gathered to explain what factors may facilitate and undermine ART adherence. However, it appears that many studies are guided by health behaviour theories, often at the expense of how behaviours such as ART adherence are enabled or supported by the wider social environments in which behavioural decisions are made. The health behaviour theories that have been used include the information, motivation and behavioural skills model (Peltzer *et al.* 2010), the health belief model (cf. Wringe *et al.*, 2009) and social cognitive theory (cf. Watt *et al.*, 2009a). All of these focus on the individual level of analysis, with behaviour viewed as the outcome of conscious rational choice. In this paper, we seek to move beyond a focus on individual behaviour shaped by cognitions such as attitudes, beliefs and motivations, to improve our understandings of how the social environment shapes the links between cognition and behaviour, with particular attention to the way in which particular forms of behaviour are enabled or limited within specific local contexts.

Some efforts have been made to examine ART adherence in relation to social contexts, for example, by using socioecological theories (Posse *et al.*, 2008; Roura *et al.*, 2009) and theories of social capital (Ware *et al.*, 2009). Ware *et al.* (2009) focused on the influence of social relations on adherence in Nigeria, Tanzania and Uganda by highlighting the role of social relationships in facilitating adherence, arguing that in conditions of chronic poverty, people on ART adhere in order to ensure that family members who support them remain motivated to do so. Although these theories help us get a little closer to understanding the behaviours that govern ART adherence, they only provide us with a snapshot of a handful of factors in any given context. It is against this background, and in view of our belief that healthcare-seeking behaviours are determined by social factors and not merely a result of individual choices, that we seek to construct an analytical framework of the spectrum of factors that influence ART adherence in Zimbabwe. In doing so, we hope to contribute to the evolving ‘fourth generation’ model of AIDS management outlined below.

## TOWARDS AN ANALYTICAL FRAMEWORK FOR UNDERSTANDING ANTIRETROVIRAL THERAPY ADHERENCE

In their review of the evolution of approaches to promoting health-enhancing HIV/ AIDS-related behaviours in the field of HIV prevention, Campbell and Cornish (2010) identify three generations of approaches, namely, information provision, peer education and community mobilisation. Currently, much emphasis is placed on the importance of community mobilisation in behaviour change efforts. Critiquing community-focused approaches as being too narrow in their scope, Campbell and Cornish (2010) argue that health-related behaviours are often shaped by many factors that lie beyond the boundaries of the local communities within which AIDS-affected people live and work. They speak of the urgent need to develop systematic accounts of the social contexts that frame community responses to health. Such a focus would constitute a ‘fourth generation’ of approaches to HIV/AIDS management, one which acknowledges the need to understand and target not only individuals and communities but also the wider social contexts in which communities are located.

Campbell and Cornish (2010) provide a preliminary conceptualisation of three dimensions of social context in which the members of AIDS-affected communities make health decisions, namely, the material, symbolic and relational dimensions of their social environments. They developed this conceptualisation in the context of the particular challenges of HIV prevention in South Africa and India, concluding that there is a need to amend and develop their account of context in a wider range of settings. In this paper, we use their framework as the starting point for our focus on AIDS treatment.

As such, we take ‘material context’ to refer to the extent to which people have access to the resources that they need to be healthy, including, for example, opportunities for paid employment, access to food aid, microcredit schemes or welfare grants. The ‘symbolic context’ consists of the meanings, the representations and the ideologies that circulate within a society, including the representations of gender and the presence or absence of AIDS stigma, which can either help AIDS-affected people cope (through enabling positive identity constructions and tolerant and inclusive social relations) or be a source of social exclusion and discrimination (Campbell *et al.* 2011c). The final dimension of context described by Campbell and Cornish (2010) is the ‘relational context’, referring to the quality of relationships and partnerships between health service users, providers and funders as well as family and community relations. It should, however, be said that all three dimensions are intrinsically linked and reflect social relations within a community. If, for example, stigmatising attitudes (symbolic) are common in a given community, this may reflect poor social relations (relational), which in turn makes it harder for people to negotiate access to support (material).

As the contexts of HIV prevention and treatment programmes differ, with, for example, treatment programmes more dependent on health facilities and nurse–patient relationships, we do not seek merely to adapt this three-level framework. Instead, we report on an empirical study that enables us to advance, expand and redevelop the framework proposed by Campbell and Cornish (2010)—specifically for the contextual analysis and action of ART adherence programmes in Africa.

## METHODOLOGY

The research question guiding this paper is: What social factors influence adherence to ART? The study from which we seek to answer this question is ongoing and was granted ethical approval from the Medical Research Council of Zimbabwe (A/681) and Imperial College London (ICREC_9_3_13). Informed and written consent was gathered from all the participants with the agreement that their identities would not be revealed. Pseudonyms have therefore been used throughout.

### Study setup and sampling

This study was conducted in the Manicaland province of eastern Zimbabwe. The impact of the AIDS epidemic on Zimbabwe has been devastating, but after peaking with an HIV prevalence rate at 26.5% in 1997, the prevalence rate has stabilised and was, in 2009, 14.3% (Zimbabwe Ministry of Health and Child Welfare, 2009). This follows reductions in high-risk behaviours between 1999 and 2004, and AIDS mortality beginning to level off (Gregson *et al.*, 2010). This study included participants from three rural sites, all of which are characterised by high levels of poverty. The majority of people living in these three communities are in informal employment or survive through subsistence farming. The three health clinics serving the communities all administer antiretroviral (ARV) drugs. The decentralisation of ART services to include even smaller rural health clinics has helped Zimbabwe provide treatment for 215 000 people (57% of those living with AIDS), and further efforts are being made to increase uptake (UNAIDS, 2010). It was through the three health clinics that we recruited our research participants. As Table 1 summarises, we recruited 25 nurses and 53 adult ARV users. The ARV users were sampled using a mix of snowball (using village community health workers), opportunistic (self-selected informants) and typical case (e.g. adherers to ART) sampling, and the nurses were recruited on the basis of their willingness to participate in the study.

**Table 1:** Summary of participants and research methods

	Participants	Interviews	FGD
Nurse	25	18	1
Patient	53	19	4
Total	78	37	5

FGD, focus groups discussions.

### Data collection and analysis

Four experienced and Shona-speaking researchers collected the data for this study in October and November 2009. As illustrated in Table 1, they conducted 37 individual in-depth interviews and five focus groups discussions, with the former lasting on an average of 1 h and the latter 2 h and 20 min. To keep the interviews focused, the local researchers used semi-structured topic guides, covering their personal background, their experiences of AIDS, stigma and ART treatment as well as the barriers and the facilitators of access and adherence to ART. The focus groups discussions not only followed a similar topic guide but also engaged the participants in a role play, demonstrating a typical patient–nurse interaction in an ART context. With their consent, we digitally recorded the interviews, allowing us to transcribe verbatim and translate the interviews into English. The transcripts were subsequently imported into the qualitative software package ATLAS.ti GmbH, Version: 6.1.12 (Berlin, Germany) for thematic content analysis (Flick, 2002).

Our analysis followed the steps of Attride-Stirling's (2001) thematic network analysis. Step 1 was to code text segments with an interpretative title. This process generated a total of ‘225’ codes. As we were not seeking to report on all of these codes, we began, guided by the conceptual framework and reviews of the literature, to explore how the codes are interconnected and identified dominant and relevant basic themes that emerged from the codes (step 2). These basic themes highlighted some of the factors influencing ART adherence. We then clustered these factors into higher-order themes, or the so-called organising themes (step 3), giving rise to the dimensions of our proposed framework. We finally clustered the organising themes into two global themes (step 4), indicating two levels of analysis, namely, contextual and psychosocial. We have illustrated this process in Tables 2 and 3, which outline each of the two levels of analysis as well as the dimensions (organising themes) that came from our interviews and their relationships. Through these steps, we have developed a data-driven conceptual framework of the contextual and psychosocial dimensions that influence ART adherence. We will now turn to describe and elaborate on the emerging themes (step 5), using the global and organising themes as the headings.

**Table 2:** Global theme: contextual level of analysis

Codes	Factors influencing adherence (basic themes)	Dimensions (organising themes)
• Poverty	1. Lack of adequate food, or fear of supplementary food costs, stop patients from taking their drugs.	Material
• Food		
• Distance to clinic		
• Transport costs	2. Patients are more likely to attend monthly consultations, and seek urgent medical treatment, if the clinics are nearby and transport is available and affordable.	
• Hospital costs		
	3. Many patients struggle to meet the hospital costs associated with AIDS treatment.	
• Stigma	4. Many patients feel stigmatised and fear being recognised as an AIDS patient, preventing them from seeking ART services.	Symbolic
• Gender roles		
• Diminishing power of traditional healers		
	5. Notions of masculinity inhibit men from participating in ART programmes and can also prevent women from adhering.	
	6. AIDS and ART are now part of the public sphere. Everyone knows how to prevent and treat AIDS.	
	7. As biomedicine proves its worth, fewer patients see the need to consult traditional healers.	
• Social support	8. Patients can optimise ART adherence by drawing on support from community members and friends.	Relational
• Children		
• Treatment partner		
• Relationship with nurses	9. Family members who act as treatment partners can facilitate ART adherence.	
	10. The quality of relationship that patients have with health professionals influences adherence.	
• Churches and faith	11. Churches can both facilitate and undermine ART adherence.	Institutional support
• Food aid/NGOs		
• Health service improvements	12. NGOs play an important role in mobilising support groups and providing food aid.	
• Counselling		
• Waiting time and opening hours	13. Good adherence depends on good quality health services, including free and readily available drugs, good counselling and advice, favourable waiting and opening times.	
• Period lack of ARVs		

ART, antiretroviral therapy; ARV, antiretroviral; NGOs, non-governmental organisations.

**Table 3:** Global theme: psychosocial dimensions influencing adherence

Codes	Factors influencing adherence (basic themes)	Dimensions (organising themes)
• Seeing improvements	14. Patients are encouraged to stay on ART when they experience improvements to health.	Patient motivation
• Side effects		
• Taking responsibility		
• Hope	15. Hope and having reasons to live a healthy life facilitate ART adherence.	
• Desire for life		
• Habit	16. With time, patients may be into the habit of taking drugs or they may become complacent and stop taking them.	
• Complacency/negligence		
	17. Patients may decide to stop taking ARVs if the side effects become too severe.	
• Disclosure and denial	18. Actively disclosing HIV status facilitates adherence as people around the patients support them.	Patient participation
• Mobilising support		
• Income generation		
• Agency	19. Some patients actively create social spaces to facilitate their adherence.	
	20. Some patients actively engage in income-generating activities to sustain the healthy diet required for ART.	
• Depression	21. Some patients, men in particular, resort to alcohol to avoid a reality as an HIV-positive individual.	Psychosocial responses to ART
• Alcohol		
• Spiritual beliefs and traditional healing	22. Patients may turn to non-biomedical resources for support, some of which may hinder or support adherence.	
• Confidence		
• Positive identities		
• Psychosocial problems	23. Many patients develop confidence in their ability to take control over their health and begin to construct positive social identities, differentiating themselves from those who have not yet been tested.	

ART, antiretroviral therapy; ARV, antiretroviral.

## CONTEXTUAL DIMENSIONS INFLUENCING ANTIRETROVIRAL THERAPY ADHERENCE

In this rural context, remarkable progress has been made in the distribution of ART. Both the patients and the nurses were very positive about the introduction and the scale up of ART; however, they also agreed that many challenges were still outstanding. ART requires much commitment and participation from patients. Once patients have tested positive and experienced some of the first and early symptoms of HIV/AIDS, they will commence the treatment process. Their CD4 count levels are measured, and they are counselled on the treatment regimen—which includes timely and daily administration of the drugs. Patients will stay on ART for the rest of their lives and are in this context required to show up for monthly review consultations where the nurses monitor the patients' progress and provide them with their monthly supply of ARVs. Although many patients in our study, in principle, were committed to adhering, a number of contextual factors shaped the likelihood that they would do so in practice. Concurring with Campbell and Cornish's (2010) framework, we found a number of material, symbolic and relational factors to both undermine and facilitate ART adherence (See Table 2). In this paper, however, we discuss the relational context from two perspectives where ‘relational’ refers to personal relationships and ‘institutional’ refers to relationships with agencies.

### Material

In the interviews, both the patients and the nurses referred to poverty as a key constraint on adherence, impacting on the patients’ ability to take their drugs in a range of different ways. Some of the more prevalent material dimensions influencing ART adherence reflect the high levels of poverty that characterise the context of this study and primarily refer to a lack of food and money compromising their treatment.

#### Inadequate access to food

A key component of the counselling and support that the nurses provide to the ARV users involves their advice on the importance of patients eating nutritional foods as part of their treatment regimen. ARVs work best if complemented with a nutrition-rich diet. However, when patients struggle to find food, and witness little progress, coupled with the discomfort that comes with taking powerful drugs on an empty stomach, some discontinued their treatment.

‘They say sometimes they don't take their tablets if they don't have something to eat… it's like there is this other female patient whom we initiated on ART, so we gave her the two weeks starter pack, and when she came back she had not taken the tablets, and we asked why, and she told us that she had not enough food to eat before taking the tablets.’ Eddy, nurse

#### Distance to clinic and transport costs

Patients who cannot afford adequate levels of food often also struggle to meet the transport costs associated with going to the health clinic to attend monthly medical reviews and to pick up their monthly supplies of drugs. This was particularly the case with patients who lived further away and who had no other choice but to walk (because of either inadequate transport infrastructure or the costs)—but often unable to find the time and energy to make the journey.

‘Distance may discourage someone to come for medication. The patient might say “a-ah I'm tired of going all the way to the hospital” “a-ah no I will go later” and the person will be defaulting.’ Paul, nurse

In conditions of poverty, some patients had to travel from place to place to search for support from various family members, which meant that it was often difficult for them to attend their monthly reviews and drug collections.

‘One patient said she had gone to Harare to visit her husband but she failed to raise the money to come back here on time for her review, so for two months she had no ARVs.’ Tonderai, nurse

An ARV distribution system that would allow patients to pick up drugs from any health facility may help those patients who are on the move.

#### Hospital costs

Aside from the costs related to getting to the clinics, the treatment itself also came at a cost. This is despite the fact that the ARVs are given for free. The three health facilities through which we recruited our informants all charged patients $1 for their monthly consultation—a cost many patients struggled to meet.

‘Some patients fail to raise money for the transport to come and collect their supplies and pay for their consultation fee. These are people who don't have any source of income. Sometimes they spend a lot of time trying to sell their produce and they might forget to take their tablets.’ Alice, nurse

‘We are unable to raise that dollar every month when we go for reviews.’ Austine, patient

Patients who struggle with food and attending monthly consultations are less likely to adhere and therefore more likely to experience opportunistic infections. Although people can have free ARVs, they have to pay for the treatment of opportunistic infections, a cost that is often so unaffordable that the patients do not see anything to be gained from seeking medical advice.

‘At times one has no money and if I know I have to pay for medicine to treat an infection that will prevent me from going to the clinic in the first place.’ Agnes, patient

In relation to material context, it was clear that poverty severely undermined ART adherence and in some cases put ART users at risk of experiencing viral replication and the advancement of AIDS (e.g. through lack of food or untreated opportunistic infections). We will discuss how some patients overcome these problems in the next section, for example, by engaging in IGAs.

### Symbolic

Various dimensions of symbolic context appeared to serve as both barriers and facilitators of adherence. The barriers discussed below include fear of being recognised as an AIDS patient, reflecting a continued presence of stigma and women's disempowered position within their household. The facilitators included the growing awareness of AIDS and ART as well as a diminishing dependence on traditional healers, whose advice was seen by many to contradict the biomedical advice that they saw as more useful in their current condition.

#### Fear of being recognised as an AIDS patient

Although many patients reported that since the advent of ART there had been a reduction in the stigmatisation of PLWHA, many patients, particularly men, spoke of feeling the fear and the embarrassment associated with being recognised as an AIDS patient, saying that stigma served as a barrier to the timely accessing of services by many men.

‘Men are generally afraid to be identified as HIV positive. They are shy and they may only “come out” after they get seriously ill. Some are afraid that people will laugh at them or look down upon them for being HIV positive.’ John, patient

Once enrolled onto ART, many men were deterred from attending the monthly consultations that required them to go to the ‘AIDS clinic’ and wait in long queues together with other AIDS patients. In such a situation, they were unable to keep their status a secret, and many found this prospect intolerable.

‘People know that this is the AIDS clinic. Some patients when they come here, they will go past the place. Then when they see me going out of the clinic, perhaps to go for tea, they will approach me secretly to ask me privately to attend to them. When I ask him to come into the clinic he will be distressed to see that there is “Mrs so and so” on the bench, whom he did not want to “come out” to.’ Claudius, nurse.

#### Women's disempowered position within households

In this context, women's social and often disempowered position stands in the way of their ability to adhere to treatment. Bridget had travelled from Manicaland to see her husband, a migrant worker lodging in Harare, to collect money for their children's school fees. However, her husband decided to spend the money on his girlfriends, refusing to give her the money she would need to travel back to Manicaland to collect her ARV treatment.

‘It was his pay day and I waited for him to come back to his lodgings in Harare after work—to give me money so that I could travel back to the rural area. However he did not come back to his lodgings. He took his money from work and went to his girlfriend's place. The second month he did the same thing. It was only in the third month that he gave me money so I could travel back. When I came back home after the third month I went to the clinic and explained to them that I hadn't been able to take my treatment for almost three months, because my husband would not give me the money to come back. The nurses were not impressed—but I was honest and they tried to help me, that's why I had to go for CD4 tests again.’ Bridget, patient.

Bridget's economic dependence on her unreliable husband highlights women's marginalised position in this context. The husbands’ unwillingness to accept their wives’ HIV status, and to support their treatment, can sometimes result in women being threatened with divorce if they insist that they are HIV positive in the face of their husbands’ disbelief and if they express determination to adhere to their ART treatment.

‘Some men refuse to believe that their wives are HIV positive. They themselves refuse to come to the clinic and get tested. Sometimes, if the wife comes here to get services, the husband will threaten to divorce the wife if she continues taking ARVs. This will affect her adherence.’ Claudius, nurse

This suggests that women's economic dependence on men, facilitated by local constructions of gender, can serve as a barrier to women's adherence.

#### AIDS and antiretroviral therapy in the public sphere

In an effort to curtail the AIDS epidemic, many information campaigns have sought to educate the general public regarding how AIDS is spread and what treatment opportunities are available— imprinting AIDS into the public sphere.

‘People now know that there is this disease. It is talked about on radio and television, people being told when to take pills and that these pills can help, that condoms can help you to prevent HIV. Condoms are encouraged on the radio, and nurses in hospitals speak about not having too many partners.’ Joe, patient

A recent WHO study suggested that 45% of Zimbabwean youth aged 15–24 years are in possession of comprehensive and correct knowledge of HIV/AIDS (WHO, 2010), testifying to the fact that the ‘wall of silence’ and denial that characterised the epidemic's earlier stages are gradually coming down, and AIDS is increasingly being acknowledged as a fact of life. This increasing social acknowledgement of the problem, coupled with the fact that contracting AIDS is no longer a death sentence, has made it easier for some people to be tested and to accept their HIV status.

‘People now have the information and are now encouraging each other to go for HIV tests. People now know that there are ARVs and if one tests HIV positive then there is hope because ARVs are now available.’ Chris, patient

As AIDS transmission and treatment technologies become part and parcel of the public sphere, people's faith in unhelpful traditional interpretations and ‘cures’ diminish.

#### Diminishing power of traditional healers

Most people in Zimbabwe have somehow been affected by AIDS and have some knowledge about the disease. This has meant that fewer people are resorting to traditional cures and explanations such as witchcraft, which were common at the earlier stages of the epidemic, and, with the introduction of ART, are appreciating the potential of biomedical technologies—giving them less reason to visit traditional healers.

‘When I first fell ill, I believed it was witchcraft, I did not know better. When I was then tested for HIV and found to be positive I felt relieved. I told my relatives not to go witch hunting if I die, knowing I will die from AIDS. Normally people would go to a witch doctor if their relative dies to find who really has caused the death. I then went through the whole process and I am now on ARVs and I am recovering well. I don't see the benefit of going to a traditional healer.’ Violet, patient

### Relational

ART adherence is heavily influenced by the social relationships that exist between ART users and the people they interact with on a daily basis, including family and community members as well as service providers. In this subsection, we highlight the important role played by social support, treatment partners and sustaining good relationships with nurses in facilitating adherence.

#### The availability of social support

In conditions of poverty, a key contributing factor to ART adherence is social support. One patient highlighted how people in his village have organised themselves so that they all support one another.

‘The best thing about my village is the way we are living. When it comes to ploughing, people share knowledge so that they can plant for themselves and sell to each other. People are encouraging each other to take their children to school. If there is anyone who is sick, and the relatives have died, people will come and help.’ Karren, patient

In addition to support from community members, the patients participating in this study also spoke about the importance of support from other ART users in providing encouragement and advice. Such support was available both through support groups and through friends.

‘We have our support group and I have a friend who always encourage me and try to make me have a positive attitude when I am down. I also do the same with fellow HIV sufferers. We try to up lift each other.’ Everline, patient

#### Family support and treatment partners

Also, family members play an integral part in a patient's adherence to ART. As the testimonies from Christine and Garikai suggest, it is crucial that family members are aware and have an understanding of their treatment regimen so that they can help remind the patient about when to take drugs and when to attend consultations.

‘My family encourages me and urge me not to miss my appointments because they now appreciate how these drugs have helped me to recover.’ Pamela, patient

‘The way some patients live, say for example they might staying with people who look down upon them, or they could be living with people who don't even take this programme seriously, so there wouldn't be anyone to remind them.’ Alice, nurse

Family members were also found to play an important role in ensuring that the patients had sufficient food and were emotionally supported.

#### Relationship with nurses

Both nurses and patients spoke about the qualities and the characteristics of a ‘good patient', indicating the importance of patients conforming to the norms of the ‘good patient persona'. This required some level of relationship building between patients and nurses, which could result in either productive and health-enabling patient–nurse relationships or a breakdown in communication, which could be detrimental to the patient who would end up fearing going to see the nurse.

‘I think a patient who follows what the nurse advised is the patient they think is good. A patient who takes all the tablets as directed and completes the course as well as coming back for reviews. A patient who adheres to what the nurses who have taught, to the nurses this will be a good patient I think.’ Albert, patient

‘A good patient will never miss their appointments or even default on their time of taking pills because they understand why they are taking those pills and they also don't want to spoil their relationship with us.’ Joe, nurse

It was evident that social relationships play a key role in facilitating or undermining ART adherence.

### Institutional support

At an institutional level, churches, non-governmental organisations (NGOs) and health services all influence a patient's ability to adhere to ART.

#### Churches influence antiretroviral therapy adherence

Our informant's had mixed responses to the role of their church in facilitating or undermining ART adherence. The Apostolic Church was often cited for ignoring the impact of AIDS and discouraging its followers to take medicines. However, not all churches and denominations were a barrier to ART. Some churches actively discouraged traditional medicines and healing and promoted AIDS testing and ARVs.

‘What discourages some people to take their tables is the church. Many churches don't encourage people to go to the hospital [… ] The church that I attend is different, they don't allow traditional medicine so I now rely on pills only.’ Karren, patient

#### Non-governmental organisations' role in mobilising support

A number of faith-based and international NGOs worked actively in the area of study. The organisations were reported to be providing people with AIDS information and to be supporting those who are already on ART. Aside from training community health workers and providing encouragement for PLWHA, NGOs were found to distribute food to ARV users—an activity that provided an incentive for non-ARV users to go and be tested.

‘I would say that what encouraged most people is that, us as black people we like food, I would say when home based care, actually AFRICARE initiated food handouts as well as Plan International, people started flocking here to come and get tested, they wanted to know whether they are HIV positive or not. Almost everyone came for testing.’ June, nurse

Many of the services provided by NGOs were co-ordinated with the local health clinic to ensure that their services reached ARV users.

#### Quality of health services

With the ARV roll-out came the training of nursing staff and a decentralisation of CD4 count machines to speed up ARV reviews. All of these, together with the availability of free ARVs, have improved people's trust and perception in the quality of healthcare services available to them.

‘Many people can now access ARVs for free. Before people were worried about the CD4 cell tests which used to be done in Mutare Town before our local clinic got the machine. Previously, patients would need to come twice before getting their results processed. This influenced the treatment of some people. But since we got the CD4 count machine we have been running smoothly.’ Colin, nurse

Having said this, despite the progress that has been made, many challenges remain, including the shortage of health staff. One patient explained how staff shortages (resulting in long waiting times) can discourage patients from going for their check-ups.

‘There are times when we feel it is impossible to see a Doctor, and one might as well just discontinue treatment and stop going there only to waste time waiting in the queue.’ Rose, patient

Other challenges faced by the health services, undermining the quality of services available to ARV users, include continued resource constraints, poor management, difficulties with repairing dysfunctional equipment, power cuts and limited access to water. This subsection has underlined some of the pathways through which the availability and quality of institutional support can influence ART adherence.

The above dimensions of context can all influence ART adherence. However, they also provide the platform through which psychosocial behaviours are enacted.

## PSYCHOSOCIAL DIMENSIONS INFLUENCING ADHERENCE

In addition to the contextual dimensions discussed above, our analysis also revealed a number of psychosocial dimensions influencing ART adherence. However, the psychosocial dimensions that influence ART adherence appear to be intertwined with the contextual dimensions discussed above. In this section, we explore different forms of behaviour influencing ART adherence but argue that they are enabled or limited by the context. As Table 3 illustrates, we have identified three broad psychosocial dimensions to influence ART adherence. The first relates to patients motivation for taking ARVs. If a context allows a patient to readily access ART and the patient experiences improved health, the patient may feel motivated to carefully adhere to the drugs. The second dimension refers to patients' interest or ability to participate in their treatment by engaging in activities that are conducive for their treatment. The third and final dimension relates to patients' psychosocial responses to ART. Depending on the context and the levels of support available, patients may engage in health-damaging activities, such as drinking alcohol, in an attempt to avoid their reality. Others may be able to construct positive social identities. We will now discuss the psychosocial dimensions in more detail, following the structure illustrated in Table 3.

### Patient motivation

How patients experience the physiological impact of ART differs—conditioned by their local context—and impacts their motivation for taking drugs. This can either facilitate (if patients experience improvements) or undermine (if patients suffer from repeated side effects) ART adherence.

#### Seeing improvements

Both nurses and patients frequently spoke about the positive impact that ART had on health and well-being of ARV users. ART has enable many patients from being weak and bedridden to being fit and productive—a change in health which motivates many to adhere.

‘I feel motivated by the mere fact that before I was on ART I was always sick and was unable to do a lot of chores but since I started taking these drugs I am fit again.’ Lucy, patient

One very visible change that ART can bring about is the weight gain of patients. In this context, looking sickly thin is associated with AIDS, and because ART can facilitate weigh gains, patients who were previously perceived as sick (and subject to stigma) now look healthy and well. The weight gain that ART can facilitate therefore influences ART adherence positively.

‘Some of them might have been motivated by the recovery they have gained. Especially those who have gained some weight think they might lose weight again if they default.’ Eddy, nurse

#### Hope and desires for life

Seeing improvements to health after being diagnosed with a disease that is often associated with death gives patients hope and a new and more positive outlook on their lives and their futures. One nurse argues that the life-prolonging opportunities that ART provide is a key reason to why some patients adhere to ART.

‘Adherence is successful when patients are taking medication so that they can live longer.’ Marta, nurse

Also related are the reasons why patients have to live a long and healthy life. Janet, for example, knows that only by adhering to ART will she stay healthy enough to care for her children and see them grow up.

‘My kids are still very young and I value my health because it's only when I am fit that I can look after my children. I still want to look after children, so I stick to what the doctor advises me to do. I keep all my review dates, and I check my diary regularly so that I don't miss any review dates.’ Janet, patient

#### Habit and complacency

How patients experience ART changes with time—changes that may either facilitate or undermine ART adherence. As Natashia highlights, many patients become used to their treatment regimen, and taking drugs becomes a habit that they do not need reminding of.

‘We get accustomed to the routine of taking those drugs’ Natashia, patient

On the other hand, you have patients who, after experiencing positive changes to their health, become complacent and believe they have recovered from AIDS.

‘I have also realized that there is also a problem with patients who would have been on ART for a long time and now feeling like they are recovered. I have discovered that they become reluctant and their rate of compliance also goes down. May be they will be feeling that they have fully recovered and have been cured, so sometimes getting ill will help them to comply.’ Esther, nurse

#### Experiencing side effects

The extent to which ART improves health differs between people. For a small minority, the health may deteriorate as a result of ART—for example, through developing Steven-Johnson syndrome (toxic epidermal necrolysis), which is a skin disease. But for the majority, discomfort arising from the physiological reactions to the powerful drugs is what makes some patients decide to discontinue their drug regimen.

‘I stopped taking ARVs because of the side effects. This is my 5th time trying these ARVs. The first time, I was on ARVs, they worked well for 3 weeks, but then some painful reactions appeared—burns started developing. That was when I stopped and was given some medication to deal with the reaction and when I was feeling better again they tried me again.’ Marie, patient

The nurses reported that the negative side effects of ART were higher amongst patients who had inadequate food. Although the side effects may only encourage patients to discontinue their treatment temporarily, it is such inconsistencies that cause drug resistance.

### Patient agency

ART does not improve health in a vacuum but reacts to the various contextual dimensions that facilitate adherence. Patients can therefore take an active and mediating role in ensuring that the context is conducive to ART adherence.

#### Disclosing their need for treatment

One important psychosocial factor facilitating ART adherence is the patients decision to disclose to others their HIV status and need for ART. Patients who tell the people close to them about their status and treatment regimen do not need to take their pills secretly—something that can result in treatment defaults.

‘There are some who still want to keep it secret that they are living with HIV. Yes, it is their decision but such people might even default because they would be trying to make sure they are not seen.’ Vincent, nurse

We have previously discussed how telling those around you, for instance, family members or those whom they live with, can be an invaluable resource for ARV users as they can provide encouragement and help remind the patients about taking the drugs and attend consultations.

#### Ability to mobilise support

In the preceding paragraphs, we have discussed the importance of social support. However, social support is not just something that is always available for patients to draw on; it requires patients to negotiate and navigate through the kinds of support that are available. Patients therefore benefit from living in a context that allows them to actively mobilise and access social support. In our context, this can be illustrated through the way in which support groups for ARV users are emerging—often as a result of their initiative and active participation.

‘HIV positive people here are organized and we have our support group. It is so encouraging to have an arrangement whereby we meet on regular basis as people living with HIV/AIDS.’ Everline, patient

In these support groups, ARV users share their knowledge and skills on activities that help them cope with the challenges to ART adherence. This includes sharing knowledge about the IGAs they do.

#### Engage in income-generating activities

Patients' engagement in IGAs signifies a number of important factors that influence ART adherence. Firstly, patients are able to engage in IGAs because of the improvements in health they have experienced as a result of ART. Secondly, IGAs provide ARV users with a platform to prove their productivity and worth to society and to develop a positive approach to being HIV positive. Finally, the IGAs allow the patients to grow healthy and nutritious foods—all of which facilitate ART adherence.

‘My HIV positive status gave me the inspiration to start participating in HIV/AIDS programmes, and I developed this positive approach, and I decided to start some income generating projects. I remember I started a poultry project and vegetable garden at our rural home which is near here. I grow carrots and have some chicken. I do this for myself so that I can eat healthy food. All these projects I started them after realizing that I am HIV positive and I would need a good diet and a little income.’ Tonderai, nurse

This subsection has highlighted the role patients can play in optimising the outcomes of their treatment. This suggests that ART-enabling contexts must be conducive and supportive of the kind of activities and participation that facilitate ART adherence.

### Psychosocial responses to antiretroviral therapy

In this final subsection, we will explore how different patients respond psychologically to their HIV status and need of treatment.

#### Drinking alcohol to avoid reality

A challenge particular to men in this context is the local constructions of masculinity, which makes it difficult for men to accept their HIV status and need for treatment. Many men fear that being HIV positive will reduce their manhood and status and therefore often resort to alcohol to avoid a reality where they see themselves to have a lesser role and purpose.

‘It is common with men… adherence is hard to achieve for men because of alcoholism. Some men will drink so much that they forget to take their drugs, and even forget that they are patients on ART.’ Colin, nurse

Although many of our male participants reported on masculinity as a barrier to ART adherence, often with reference to alcohol use, they themselves had overcome these barriers—suggesting that far from all men struggle to come to terms with their HIV status and need to adhere to ART.

#### Spiritual advice and traditional healers

Some patients resort to the spiritual world for advice and to give meaning to changes in their lives. Although some churches are unhelpful in providing support to ART users, many patients still look towards faith to find encouragement and meaning to difficult circumstances—providing them with positive attitudes and outlook on life, which can facilitate ART adherence.

‘It helps as it enables a person to think positively because the biblical advice that you get and the encouragement that you are not the only one in this situation you notice that it gets rid of the negative thinking that you had that might have deteriorated your health or your life you see.’ Nicola, patient

Others may use faith and spiritual advice differently, hoping that it can provide a cure for AIDS. Such patients, as one nurse illustrates, are more likely to periodically discontinue their treatment—presenting a barrier to ART treatment.

‘This one ART patient of mine decided to consult a traditional healer, when she got there she was told that she was not HIV positive and she should stop taking those ARVs and was given some concoction, that she was told would cure her illness all together. So she stopped coming here, and when she came here she had deteriorated so much.’ Esther, nurse

#### Constructing empowering social identities

The availability of ART has provided some patients with a renewed and empowering identity—one that draws on the health gains they have experienced as a result of ART, which translates AIDS from a death sentence to a chronic and manageable disease. Through an ‘us and them’ distinction, a number of ARV users highlighted themselves as responsible citizens who are now worthy of respect from ‘the other’.

‘Now people with HIV are able to feel like any other fit person… these drugs have removed signs and symptoms of HIV and made us healthy again, gaining the respect of other people in the community who would have condemned us to death. It has made us fit again, as you might as well know we could have died or been bedridden by now. The drugs have brought life and restored dignity to us. People no longer look down upon us that much.’ Pamela, patient

This subsection has explored some of the representations and the meanings that ARV users draw on as a response to their ART treatment. Depending on the individual patient, these representations and meanings can facilitate or undermine their adherence to ART and are worthy of our attention.

## DISCUSSION

A great number of studies have explored the structural and behavioural barriers and facilitators to successful ART adherence. They have provided us with very useful snapshots of the determinants of successful treatment compliance in various contexts. Many of the barriers or the facilitators we refer to here have been identified in other contexts. These include lack of adequate food, transport costs, waiting times, side effects and fear of stigma (Mshana *et al.*, 2006; Hardon *et al.*, 2007; Murray *et al.* 2009) as well as the role of alcohol, family members and treatment partners, social support and personal motivation to treatment (Dahab *et al.*, 2008). However, our analysis has revealed the existence of several additional factors including the role of patient/nurse relationships as well as gendered positions in facilitating or undermining ART adherence. We are reporting on these factors in greater detail elsewhere (Campbell *et al.*, 2011a, Skovdal *et al.*, in press, Skovdal *et al.*, 2011).

Rather than providing another study highlighting a limited area of factors influencing ART, we have in this paper, as the first of its kind in Zimbabwe, presented a broad spectrum of barriers and facilitators to ART adherence, all of which can tell us a great deal about how we can improve compliance to AIDS treatments. Informed by ‘the health-enabling social environment’ model, we have highlighted the networks and the relationships between some of the most prevalent influences to ART adherence in our case study. We did this with the aim to develop a framework that can assist with the analysis, planning and execution of ART programmes in other African contexts. As detailed by the structure of our discussion of findings, the framework we have developed consists of four contextual and three psychosocial dimensions. Table 4 summarises our proposed analytical framework.

**Table 4:** Analytical framework to explore factors influencing ART adherence

Influences on ART						

Contextual dimensions				Psychosocial dimensions		
	
Material	Symbolic	Relational	Institutional support	Patient motivation	Patient participation	Psychosocial responsesto ART
For example						
• Access to food	• Fear and stigma	• Social support	• Support from churches	• Seeing improvements	• Status and need for treatment	• Alcohol abuse
• Distance to clinic	• Gender constructions	• Family support and treatment partners	• Support from NGOs	• Hope and desire for life	• Mobilising social support	• Spiritual advice
• Transport costs	• AIDS and ART in public sphere		• Quality of health services	• Habit and complacency		• Constructing empowering social identities
• Treatment related costs	• Influence of traditional healers	• Patient/nurse relationships		• Experiencing side effects	• Engaging in income-generating activities	

ART, antiretroviral therapy; NGOs, non-governmental organisations.

Through this framework, we propose that attention should be given to the contextual and psychosocial dimensions that can influence successful compliance to treatment. We thereby provide ART programme planners and implementers with a tool that encourages them to analyse the content of each of the seven dimensions outlined in Table 4. The content of the contextual and psychosocial dimensions will differ between socio-economic and cultural settings; however, our study, and the numerous other studies that have looked at ART adherence in Africa, provided useful pointers towards what the content of the seven factors that we have identified to influence ART adherence might be.

In our case study, the *material context* refers to how poverty and sources of economic and materialistic support influence patients' ability to adhere to ART. In our study, this translated into lack of food and difficulties meeting the costs associated with treatment. In agreement with a recommendation made by Adato and Bassett (2008), these findings suggest that there is an urgent need to include ARV users in social protection schemes such as food and cash transfers. The *symbolic context* highlights some of the meanings and the representations that flourish in a given context and influence ART adherence. Although we found that widespread and improved knowledge of AIDS (highlighting the importance of information) has diminished the role of traditional healers, gendered stereotypes and fear of stigma still undermined ART adherence of men in particular. These findings have highlighted the importance of creating social spaces that can challenge and strengthen meanings and representations in such a way that patients begin to ascribe positive value and meaning to otherwise difficult circumstances (Goudge *et al.*, 2009; Campbell *et al.*, 2011b). The *relational context* refers to social relations, both within and between families, community groups and organisations. Although this may also include forms of leadership, our case study was limited in scope to only include the relations between the patient and his or her social environment, including family and community members and nurses at the hospital. The influence of social relations on ART adherence was the focus of a study by Ware *et al.* (2009) who argue that social relations can explain the favourable ART adherence rates found in Africa. Needless to say, the *institutional context* plays a fundamental role, particularly when it comes to the availability and the quality of health services. Numerous studies have explored how the quality of health services influence ART uptake and adherence (McCoy *et al.*, 2005; Kip *et al.*, 2009; Nsigaye *et al.*, 2009). However, as illustrated from our case study, other institutional actors that may influence compliance to AIDS treatment include NGOs and churches. Our study highlighted different aspects to *patient motivation*, underlining the importance of its role as a psychosocial dimension to successful treatment compliance. What motivates patients to (dis)continue taking medication differs and happens at a reflexive and non-reflexive level. At the reflexive level, patients are motivated by seeing improvements to their health (value of treatment) and how this positively impacts on their role as wives, husbands or parents. On the other hand, patients may be demotivated by the side effects that may arise as a result of the medication. At a non-reflexive level, patients may get into a habit of taking their medication, or they may be complacent and thereby discontinue taking their medication. *Patient participation* highlights the different initiatives that patients engage in, in interaction with their local context, to achieve optimal health outcomes. Such initiatives include disclosing their status and need for the ART as well as their participation in support groups and income-generating projects. Although the need for ARV users to disclose their HIV status has been discussed elsewhere (Sanjobo *et al.* 2008), little attention has been given to the active participation of patients in sustaining their treatment. In a forthcoming study, we discuss how patients negotiate a ‘good patient’ persona in order to achieve the best possible care from nurses (Campbell *et al.* 2011a). The final psychosocial dimension of this framework refers to the patient's *psychosocial responses to ART* and involves the different ways in which patients react and respond psychologically to the treatment regimen. Again, we found the patients to respond differently. Some patients, particularly men, sought to avoid the perceived grim reality of having lost their manhood to AIDS, repeatedly reminded to them by their daily drugs, by excessive drinking of alcohol. On the other hand, men and women were found, through social spaces and with each other's help, to have developed new and empowering identities that help them give meaning and purpose to life.

Several potential limitations of this study exist. The framework we have presented in this paper is by no means complete, and we encourage other researchers to both explore the applicability of our framework and develop it further, with other demographic groups and geographical settings—adding on to our contextual and psychosocial levels of analysis. We are also not saying that the content of the dimensions will be the same in every context. Given that our data were collected mainly in rural areas of eastern Zimbabwe with Shona-speaking participants, we concur with Flyvbjerg (2001) that the generalisability of findings to other locations should be established on a case by case basis. Nevertheless, although the content of the dimensions presented in this paper will differ between contexts, we believe that the framework provides a useful starting point for programme planners to examine the influences that may compromise or facilitate successful compliance to ART in any resource-poor context.

## CONCLUSION

In summary, this study has reported on some of the more prevalent barriers and facilitators to ART adherence in Zimbabwe and classified them into a framework that is transferrable for the analysis and action of ART programmes elsewhere. The framework argues that ART programme planners and implementers need to pay attention to four contextual (material, symbolic, relational and institutional) and three psychosocial (patient motivation, patient participation and psychosocial responses to ART) dimensions in their analysis of factors that may facilitate or undermine successful compliance to AIDS treatment in Africa.
